# Nanoplatforms for the Delivery of Nucleic Acids into Plant Cells

**DOI:** 10.3390/ijms242316665

**Published:** 2023-11-23

**Authors:** Tatiana Komarova, Irina Ilina, Michael Taliansky, Natalia Ershova

**Affiliations:** 1 Shemyakin-Ovchinnikov Institute of Bioorganic Chemistry, Russian Academy of Sciences, 117997 Moscow, Russia; irinailina.bio@gmail.com (I.I.); michael.taliansky@mail.ru (M.T.); ershova@vigg.ru (N.E.); 2 Belozersky Institute of Physico-Chemical Biology, Lomonosov Moscow State University, 119991 Moscow, Russia; 3 Vavilov Institute of General Genetics, Russian Academy of Sciences, 119333 Moscow, Russia

**Keywords:** nanocarrier, RNA interference, topical dsRNA delivery, spray-induced silencing

## Abstract

Nanocarriers are widely used for efficient delivery of different cargo into mammalian cells; however, delivery into plant cells remains a challenging issue due to physical and mechanical barriers such as the cuticle and cell wall. Here, we discuss recent progress on biodegradable and biosafe nanomaterials that were demonstrated to be applicable to the delivery of nucleic acids into plant cells. This review covers studies the object of which is the plant cell and the cargo for the nanocarrier is either DNA or RNA. The following nanoplatforms that could be potentially used for nucleic acid foliar delivery via spraying are discussed: mesoporous silica nanoparticles, layered double hydroxides (nanoclay), carbon-based materials (carbon dots and single-walled nanotubes), chitosan and, finally, cell-penetrating peptides (CPPs). Hybrid nanomaterials, for example, chitosan- or CPP-functionalized carbon nanotubes, are taken into account. The selected nanocarriers are analyzed according to the following aspects: biosafety, adjustability for the particular cargo and task (e.g., organelle targeting), penetration efficiency and ability to protect nucleic acid from environmental and cellular factors (pH, UV, nucleases, etc.) and to mediate the gradual and timely release of cargo. In addition, we discuss the method of application, experimental system and approaches that are used to assess the efficiency of the tested formulation in the overviewed studies. This review presents recent progress in developing the most promising nanoparticle-based materials that are applicable to both laboratory experiments and field applications.

## 1. Introduction

Recent progress in nanomaterials research has paved the way to the utilization of nanocarriers for intracellular delivery of different cargo. This ground-breaking biotechnological approach could be used both for solving fundamental scientific problems and development of techniques for sustainable agriculture. Nanocarriers are systems that load cargo incorporated into organic or inorganic matrixes and have a size between 10 and 1000 nm. Nanocarriers have been extensively studied and successfully applied to animal cells (reviewed, for example, in [1,2,3]) while plant cells are much more complicated objects for intracellular delivery due to the presence of additional physico-chemical and mechanical barriers (cuticle, cell wall, acidic environment, etc.) to penetration compared to animal cells [4]. Nevertheless, the number of studies demonstrating efficient nanoparticle-based delivery of various cargo into plant cells is growing, which opens up a perspective for the development of new-generation technologies for sustainable agriculture, allowing the improvement of the productivity and disease resistance of crops without modification of their genomes and in an environment-friendly way [5].

**Figure 1:**
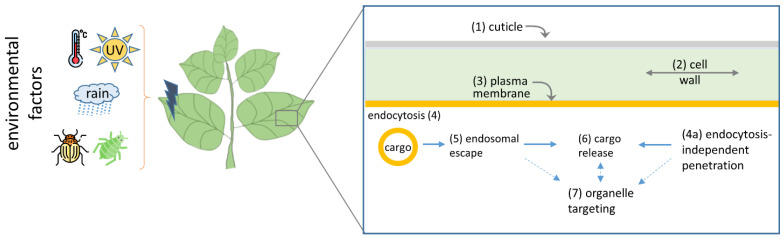
Mechanical barriers and other hurdles to the foliar uptake and delivery of the cargo into plant cell. Multiple environmental factors such as UV radiation, high temperature, rain, mechanical damage, pests, etc. could decrease the efficiency of nucleic acid foliar uptake, affecting its stability and integrity. Leaves are covered with a wax protective layer: cuticle (1). The second barrier is a rigid and viscose cell wall (2). The third barrier is a plasma membrane (3), which could be penetrated via endocytosis (4) or in an endocytosis-independent way (4a). Endocytic vesicles deliver their cargo to the early endosome, ending up in the vacuole; thus, the problem of endosomal escape (5) has to be overcome. Timely cargo release (6) from the complex with nanoplatforms should also be taken into account. If cargo is to be delivered to a particular cellular compartment, it should be targeted there using a special signal (7) that is contained in the nanocarrier or in the cargo itself.

Plant treatment using dsRNA/siRNA allows inducing transient changes in the phenotype and activating specific defense responses against particular pathogens. This approach is based on RNA interference (RNAi), a common mechanism for plant and animal cells aimed to suppress foreign or regulate endogenous gene expression at the RNA level [6,7,8,9,10]. RNAi is widely used both for fundamental science and for biotechnological tasks [11]. The induction of RNAi using plant foliar treatment with a solution of naked dsRNA/siRNA is an appealing tool that could be used for protection against viruses, fungi and pests (reviewed in [11,12,13,14,15]). However, the efficiency of RNA penetration into plant cells is questionable because (1) naked dsRNA/siRNA molecules are rather unstable in the environment and are subjected to nuclease digestion, and (2) the intracellular uptake of RNA sprayed onto the leaf surface is rather low due to multiple physical and chemical barriers (Figure 1). Naked nucleic acid applied to the leaf surface suffers from the impact of environmental factors: UV radiation, heat, rain and enzymatic and chemical damage [16]. Another challenge is nucleic acid’s penetration into plant cells. The major mechanical barriers are the cuticle and cell wall. The aerial parts of the plant are covered with a lipophilic coating, the cuticle, that consists mainly of cutin and wax and protects the plant from water losses and environmental stresses. Its permeability depends on the charge and size of the penetrating molecules, as well as temperature and humidity [17,18]. The cell wall represents another significant barrier for intracellular cargo delivery. It contains a pectin matrix, cellulose and hemicellulose fibrils and a protein component [19]. The cell wall thickness varies from several hundred nanometers to several microns [20]; its permeability depends on the cell wall pore size [21]. From the apoplast, cargo internalization occurs via endocytosis or in an endocytosis-independent way (Figure 1).

Nucleic acid penetration across two main mechanical barriers, the lipophilic cuticle and rigid cell wall, is extremely difficult without additional aid, for example, leaf surface abrasion, high-pressure bombardment, infiltration or treatment using special chemicals [4,9,10]. Two main traditional approaches to nucleic delivery into plant cells are *Agrobacterium*-mediated transformation and the biolistic method [5,22]. But both approaches have significant limitations. The Agrobacterium-based technique is not suitable for most plant species, while biolistic delivery requires special equipment and results in plant tissue damage. Nanocarrier-mediated delivery represents an approach lacking the abovementioned limitations, being more universal and allowing precise intracellular targeting. Moreover, nanomaterials are able to provide protection of the nucleic acid from environmental factors [23,24], which is very important, especially in the case of RNA, and to increase the efficiency of its penetration into the cell.

**Figure 2:**
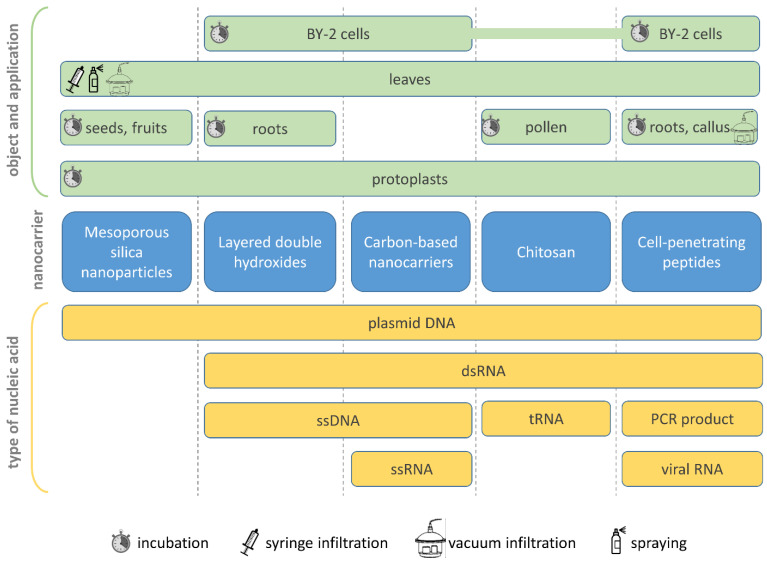
Schematic representation summarizing data on selected nanoplatforms obtained in different plant experimental systems and with various cargo.

Various experimental systems are used to assess the suitability of particular nanocarriers (Figure 2, Table 1). Protoplasts, similar to animal cell culture, lack cell wall [25] and represent a convenient experimental system for the initial tests that could be performed during the preliminary evaluation and screening of novel nanocarriers. For example, the ability of nanocarriers to cross the plasma membrane or the toxicity of the nanomaterial could be assessed. Another model system suitable for screening is a suspension culture of plant cells (in particular, *Nicotiana tabacum* BY-2 cells) possessing cell wall, thus allowing the assessment of nanoparticles’ ability to cross one of the main barriers: the cell wall. However, the results obtained in these two systems should be then confirmed for the whole plant or at least for its parts. At this stage, biotoxicity and biocompatibility issues arise more acutely, as well as the complex effect on the plant in general. Initial studies of each nanocarrier in laboratory conditions often include such plant treatments as leaf abrasion or leaf infiltration, abaxial stomata flooding, root soaking or infiltration and petiole adsorption (Figure 2, Table 1). These methods load nanoparticles into the intercellular space or vascular system, followed by intracellular cargo delivery [9,10]. But the final aim is to develop a formulation for foliar topical spraying to make it suitable for agricultural applications.

Cargo internalization could be confirmed using fluorescent microscopy; however, additional markers should be used to distinguish apoplast delivery from cytoplasmic localization [26]. Of note, the most convincing evidence of successful delivery is functional tests based on (1) reporter gene expression in case of pDNA or PCR product delivery [27]; (2) visible phenotypic changes induced by the silencing of endogenous plant genes (e.g., magnesium chelates, phytoene desaturase) by the delivered dsRNA/siRNA [28,29]; (3) silencing of transgenes in a stably transformed plant expressing genes of fluorescent proteins [26]; (4) activation of RNAi resulting in resistance to fungal or viral pathogens [23].

Despite the desirable molecule usually being RNA (dsRNA/siRNA), many studies use a pDNA or PCR product to confirm the applicability of the studied formulation because DNA is more stable, easier to manipulate and more convenient for conducting functional tests compared to RNA. Moreover, the detection of encoded by pDNA reporter gene expression unambiguously indicates efficient pDNA intracellular uptake. Moreover, pDNA could be used not only as a “model molecule” for nanocarrier-mediated delivery but a valuable tool per se as it allows (1) the transient expression of resistance genes operating against various pathogens [30] or artificial micro RNA against viruses [31], and (2) transformation of non-model plants that are not susceptible to Agrobacterium-mediated gene transfer. Efficient pDNA intracellular delivery has great potential as a laboratory approach to studies in functional genomics as it represents an alternative to Agrobacterium and chemical plant transformation, which is per se a stress factor for a plant. 

Nucleic acid delivery into plant cells could be applied to searching for host resistance [32,33,34,35] or susceptibility factors [36,37,38]. Solving fundamental problems in the field of plant–pathogen interactions paves the way to the transfer of the developed technologies to the applied biotechnology for crop defense and improvement, especially using such instruments as RNAi [12].

Advances in the development and utilization of various nanocarriers are reflected in numerous emerging studies on successful RNA or pDNA delivery into plant cells (Table 1). Nanocarriers partially solve the problems connected with nucleic acid (especially RNA) stability and delivery into plant cells. Nucleic acid being adsorbed on a nanoparticle or incorporated into it becomes less susceptible to environmental factors and crosses plant cell barriers toward the cytoplasm more efficiently. Moreover, nanocarriers could mediate gradual release and cargo penetration through the cuticle and cell wall into the cytoplasm. In this review, we are focusing on those nanoplatforms that were demonstrated to be successfully used for nucleic acid delivery into plant cells and even into different compartments of the cell. We paid special attention to the experimental systems (from protoplasts and suspension cultures to whole plants), application methods (infiltration, incubation, spraying, etc.) and, finally, approaches used for delivery confirmation (microscopy, gene expression assays and functional tests). We consider recent progress in nanocarriers’ application to the intracellular delivery of nucleic acids and summarize the data on the most prospective nanoplatforms with major potential for practical applications.

**Table 1:** Nanoplatforms and their application to plant experimental systems.

Nanoplatform	Cargo or Label	Object (Cells, Plants, etc.)	Application Method	Analysis Technique, Functional Tests	Reference
Mesoporous silica nanoparticles (MSN)					
FITC- or RITC-doped MSNs functionalized with APTMS, TMAPS, THPMP	pDNA	*N. tabacum* protoplasts, *A. thaliana* roots	Root incubation in 1/2 MS media supplemented with MSN for 24 h or with MSN/DNA complex for 48 h	FITC- or RITC-labeled MSN intracellular uptake was confirmed using fluorescent microscopy; MSN/DNA complex internalization shown using confocal microscopy (fluorescence) and transmission electron micriscopy (TEM) (immunogold) of mCherry expressed from the delivered DNA	[39]
FITC- or β-oestradiol-doped MSNs, gold-capped MSNs	pDNA	*N. tabacum* protoplasts, *Zea mays* embryos	Protoplast incubation with MSNs; tissue bombardment	MSN/DNA complex penetration confirmed using fluorescent microscopy (FITC monitoring or GFP detection) or TEM (for gold-capped MSN)	[40]
~40 nm APTES-functionalized MSNs	pDNA	*S. lycopersicum* leaves	Spraying the abaxial surface of leaves; injection into the shoot or leaves	Intracellular delivery of pDNA confirmed using RT-PCR and detection of β-glucuronidase (GUS) activity in leaves or protection against *Tuta absoluta* when cry1-encoding pDNA was delivered. Injection of the shoots is not effective. Leaf injection was demonstrated to be more efficient than spraying	[41]
FITC- or RITC-doped MSNs	No cargo	*A. thaliana* protoplasts, *Z. mays*, *Triticum aestivum* and *Lupinus* sp. roots	Root incubation in the MSN solution	Penetration of the FITC- or RITC-labeled MSNs into the cell wall and vasculature confirmed using TEM and fluorescent microscopy	[42]
MSN–APTES–FITC	No cargo	*A. thaliana*, *T. aestivum* seeds, *Lupinus* sp.	Vacuum infiltration of *A. thaliana* seedlings, lupin root incubation, wheat seed germination in growth medium supplemented with MSNs	Root uptake, presence in the intercellular space after vacuum infiltration and cellular uptake were confirmed using fluorescent microscopy	[43]
APTES-functionalized MSNs	pDNA	*S. lycopersicum* fruits at early ripening stage	Injection into fruits	The successful delivery of pDNA in a APTES-MSN/DNA complex into seeds of ripening fruits was confirmed by obtaining stably transformed plants after the germination of these seeds	[44]
Layered double hydroxides (LDHs)					
LDH-lactate nanosheets	FITC-, TRITC-conjugated LDH, ssDNA–FITC	BY-2 cells, *A. thaliana* seedling roots	Co-incubation	Microscopy Fluorescent dyes penetrated cells even in the presence of endocytosis inhibitors	[45]
50 nm LDH nanoparticles	FITC-labeled LDH, dsRNA–Cy3, dsRNA	*S*. *lycopersicum* developing pollen	Co-incubation	Microscopy of FITC-labeled LDH, dsRNA–Cy3, functional testing, transgene (GUS) silencing	[46]
40 nm LDH nanoparticles	siRNA, Cy5-labeled 21-bp DNA, siRNA	*N. benthamiana*, *A. thaliana*, *T. aestivum* leaves	Infiltration	Confirmed leaf cell penetration, apoplast and vasculature distribution, microscopy and functional tests, silencing of the transgene (16C line)	[47]
BioClay (LDH sheets)	dsRNA	*N. tabacum*, *Vigna unguiculata* leaves	Topical application, spraying	Prolonged effect: dsRNA detected on the leaf surface for up to 30 days; microscopy of Cy3-labeled dsRNA and functional tests confirmed antiviral (CMV, PMMoV) effect	[23]
Colloidal LDH nanosheets	dsRNA	*S. lycopersicum* leaves, roots, fruit; *Fusarium oxysporum*	Spraying on plant leaves, leaf petioles adsorption, dipping the plant roots; in vitro solution application on *F. oxysporum* micelium	In vitro antifungal activity of the LDH–dsRNA on mycelial growth and virulence; silencing of essential *F. oxysporum* genes using LDH–dsRNA; topical spraying provided protection from Fusarium crown and root rot for up to 60 days	[48]
LDH nanosheets	YOYO-labeled pDNA, pDNA encoding artificial microRNA	*S. lycopersicum*, *N. benthamiana* leaves, *Allium cepa* epidermis	Spraying plant leaves with an atomizer	Confirmed delivery of pDNA labeled with YOYO-1 dye into onion epidermis and *N. benthamiana* leaf cells using microscopy; systemic transport of pDNA-YOYO was observed up to 35 days after treatment in *N. benthamiana* and *S. lycopersicum*. Tomato yellow leaf curl virus (TYLCV) challenge was performed on plants pre-treated with pDNA–LDH: increased resistance of pre-treated plants was observed during 35 days	[31]
Carbon dots (CDs) and single-walled carbon nanotubes (SWNT)					
PEI-functionalized CDs	pDNA	*O. sativa*, *T. aestivum*, *Phaseolus radiatus* leaves and *O. sativa* roots	Wheat leaf topical application (twice a day), rice seedling root soaking, vacuum infiltration of rice calli	Expression of the pDNA-encoding genes (hygromycin resistance, GUS enzymatic assay, eGFP and mCherry fluorescence microscopy)	[49]
PEI-functionalized CDs	dsRNA, FITC-labeled dsRNA	*Cucumis sativus* seedlings	Spraying under pressure of 2.5 bar	Fluorescent microscopy of FITC-labeled dsRNA; qRT-PCR	[50]
PEI-functionalized CDs	siRNA (22-mer)	*N. benthamiana* 16C line leaves; wild-type *N. benthamiana*; transgenic GFP-expressing *S. lycopersicum*	Topical application via spraying in presence of 0.4% nonionic surfactant	Systemic silencing of (i) GFP in 16C *N. benthamiana* or transgenic *S. lycopersicum* plants and (ii) endogenous *CHLH* and *CHLI* genes encoding the H and I subunits of magnesium chelatase. Confirmed using visualization and qRT-PCR	[29]
PEI/PEG-functionalized CDs	dsRNA, FITC-labeled chitosan and Cy3-labeled dsRNA	*N. benthamiana*	Leaf infiltration and spraying, root soaking	Confocal microscopy of labeled dsRNA and nanoparticles; functional tests confirming antiviral effect against PVY (qRT-PCR and Western blotting); miRNA sequencing confirming RNA interference induction	[51]
SWNTs, SWNT/FITC	ssDNA, FITC-labeled DNA	*N. tabacum* BY-2 cells	Co-incubation	Fluorescent microscopy of SWNT/FITC and SWNT/DNA–FITC complexes	[52]
PEI-SWNTs	pDNA	*O. sativa* leaves and embryos	Infiltration	qRT-PCR analysis, GFP and YFP confocal imaging, GUS histochemical test, PDS knock-out phenotype observation	[28]
PEI-SWNTs	Cy3-tagged pDNA, pDNA	Wild-type and transgenic mGFP5 *N. benthamiana*, *Eruca sativa*, *T. aestivum* and *Gossypium hirsutum* leaves, *E. sativa* protoplasts	Leaf infiltration, protoplasts co-incubation	Transmission electron microscopy and direct near-infrared imaging, confocal microscopy, qRT-PCR analysis of pDNA expression, droplet digital PCR	[53,54]
SWNT-PM-CytKH9, SWNT-PM-KH9	SWNT-PM conjugated with CytKH9 peptide labeled with DyLight488 fluorescent dye, pDNA	Seven-day-old *A. thaliana* seedlings, roots	Vacuum/pressure infiltration	Confocal laser scanning microscopy, confocal Raman microscopy, Western blotting, luciferase activity assay	[55]
Chitosan–SWNT	pDNA, Cy3-labeled DNA	*E. sativa*, *Nasturtium officinale*, *N. tabacum*, *Spinacia oleracea* plants and *A. thaliana* protoplasts	Co-incubation with protoplasts; whole plant leaf infiltration	Confocal microscopy, detection of near-infrared fluorescence of SWNT and YFP expressed from pDNA	[56]
Chitosan					
TPP crosslinked chitosan	FITC-labeled BSA and Cy3-labeled tRNA	*N. benthamiana* leaves	Syringe leaf infiltration	Confocal microscopy	[57]
TPP crosslinked chitosan	Cas9 endonuclease in complex with guide RNA	*S. tuberosum* apical meristem	Vacuum infiltration	Gene (coilin or phytoene desaturase) editing confirmed by sequencing and RT-PCR	[58,59,60]
N-2-hydroxypropyl trimethyl ammonium chloride chitosan (HACC)	pDNA, FITC-labeled HACC	*N. benthamiana* leaves	Syringe leaf infiltration	Confocal microscopy, functional tests on antiviral resistance	[61]
HACC	dsRNA, FITC-labeled chitosan and Cy3-labeled dsRNA	Laboratory experiments: *A. thaliana* protoplasts, *N. benthamiana* plants Field experiments: *N. tabacum*, *S. lycopersicum*, *Capsicum annuum*	Leaf infiltration and spraying, root soaking	Confocal microscopy of labeled dsRNA and nanoparticles; functional tests confirming antiviral effect against PVY (qRT-PCR and western-blot); miRNA sequencing	[51]
HACC	dsRNA, FITC- and Cy3-labeled dsRNA	*A. thaliana* protoplasts, *N. tabacum* plants and pollen	Leaf infiltration and spraying, pollen co-incubation, root soaking	Confocal microscopy of labeled dsRNA and nanoparticles; functional tests confirming antiviral effect against TMV (qRT-PCR and western-blot); siRNA sequencing confirming RNA interference induction	[62]
Cell-penetrating peptides (CPPs)					
CPP from capsid proteins of plant viruses: BMV, BYDV, TCSV, BeYDV	FlAsH dye, BMV RNA, dsRNA	*A. thaliana* protoplasts and seedlings; *Hordeum vulgare* protoplasts, roots and mesophyll	Protoplast incubation, seedling and root soaking	Protoplast and root uptake was confirmed by microscopy, Western- and Northern-blot, qRT-PCR	[63]
Tat and its doubled variant Tat_2_, transportan, pVEC	CF-labeled CPPs	Triticale mesophyll protoplasts, *A. cepa* epidermal cells, leaf bases and root tips of seven-day old triticale seedlings	Protoplast incubation, root soaking	Protoplast and root uptake was confirmed by microscopy and fluorimetric analysis	[64,65]
Tat and its doubled variant Tat_2_, transportan, pVEC	GUS, pDNA	*T. aestivum* immature embryos	Embryos were permeabilized with toluene ⁄ ethanol and incubated CPP or CPP/cargo complexes solution	Fluorescent microscopy, GUS histochemical tests	[66]
Arginine-rich peptides (R9, R12)	Cy3-labeled pDNA, R9-GFP fusion protein, FITC-labeled dsRNA (0.9 or 0.4 kb)	*V. radiata* and *Glycine max* roots; *A. cepa* and *S. lycopersicum* roots; *N. tabacum* suspension culture	Roots incubation in the solution of R9-GFP or R9/pDNA-Cy3 complex; suspension culture incubation with R12/dsRNA complexes	Fluorescent label internalization was confirmed by microscopy; R9/pDNA and R12/dsRNA complex formation confirmed by gel retardation assay; dsRNA internalization induced silencing of transgene in suspension culture	[67,68,69]
55 CPP library	TAMRA-labeled CPP	BY-2 cells, leaves of *N. benthamiana*, *A. thaliana*, *S. lycopersicum*, poplar, and *O. sativa* callus	Incubation with BY-2 cells, leaves infiltration, rice callus was treated with CPP solution	TAMRA-CPP cellular uptake confirmed by confocal microscopy	[70]
Synthetic CPPs combining either amphipathic BP100 peptide or Tat peptide with polycationic peptide (Lys/Arg/His in different combinations)	pDNA, Cy3-labeled pDNA	*N. benthamiana* and *A. thaliana* leaves	Infiltration	Registration of protein products synthesized from plasmid pDNA–luciferase and GFP; microscopy of Cy3-labeled pDNA intracellular distribution	[27]
BP100CH_7_ (with -S-S- bonds)	pDNA, Cy3-labeled pDNA	*A. thaliana* leaves	Infiltration	Fluorescent microscopy, luciferase activity assay	[71]
BP100(KH)_9_, BP100CH_7_ (with -S-S- bonds)	Citrine yellow fluorescent protein	*O. sativa* callus	Vacuum infiltration	Fluorescent microscopy, western-blot	[72]
(KH)_9_-BP100	Cy3-lableled dsRNA	*A. thaliana* leaves	Infiltration	Fluorescent microscopy, local silencing of YFP-encoding transgene	[26]
MAL-TEG-based micelles decorated with CPP (Tat, BP100 or KAibA peptide) and EDPs	pDNA, Cy3-labeled pDNA	*A. thaliana* seedlings	Vacuum infiltration	Fluorescent microscopy: Cy3-pDNA or GFP produced from pDNA; luciferase activity assay	[73,74]
BP100 conjugated with cationic peptides; CPPs with chloroplast-targeting signal	Cy3-labeled pDNA, dsRNA, siRNA	*A. thaliana*, *G. max*, *S. lycopersicum*, *N. tabacum* leaves	Topical application via spray	Fluorescent microscopy: Cy3-labeled DNA internalization, transgene (GFP, YFP) silencing by siRNA or dsRNA; GUS histochemical tests (expression from the plasmid); in chloroplasts-luciferase activity assay (expression from the plasmid), chloroplast transgene (GFP in transplastomic tobacco) silencing by siRNA	[75]
CPPs with chloroplast-targeting signal (AtOEP34)/mixture of CPP and CTP peptides	pDNA, siRNA,	*N. tabacum*, *O. sativa*, *A. thaliana* and *N. benthamiana* leaves, tomato fruit and roots; *S. tuberosum* tubers	Topical application via spray, leaf and tomato fruit infiltration, tomato roots and potato tubers vacuum infiltration	Fluorescent microscopy, luciferase assay, western-blot; chloroplast genome integration via homologous recombination confirmed by Southern-blot and reporter gene expression	[76,77,78,79]
CPPs with mitochondria-targeting signal	pDNA	*A. thaliana* leaves and seedlings; *N. tabacum* seedlings	Infiltration, vacuum infiltration	Fluorescent microscopy, detection of reporter genes expression: luciferase assay, western-blot; mitochondrial genome integration via homologous recombination confirmed by Southern-blot and reporter gene expression	[76,80]

FITC—Fluorescein isothiocyanate; RITC—Rhodamine B isothiocyanate; APTMS—3-aminopropyltrimethoxysilane; TMAPS—N-trimethoxysilylpropyl N,N,N-trimethylammonium chloride; THPMP—(3-Trihydroxysilyl)propylmethylphosphonate; pDNA—Plasmid DNA; TEM—transmission electron microscopy; GUS—β-glucuronidase; APTES—Aminopropyl triethoxysilane; TRITC—Tetramethylrhodamine isothiocyanate; CMV—Cucumber mosaic virus; PMMoV—pepper mild mottle virus; TYLCV—Tomato yellow leaf curl virus; PEI—Polyethylenimine; PEG—Polyethylene glycol; PVY—Potato virus Y; PDS—Phytoene desaturase; TPP—Tripolyphosphate; HACC—N-2-hydroxypropyl trimethyl ammonium chloride chitosan; TMV—Tobacco mosaic virus; BMV—Brome mosaic virus; BYDV—Barley yellow dwarf virus; TCSV—Tobacco curly shoot virus; BeYDV—Bean yellow dwarf virus; Tat—HIV-1 Tat basic domain peptide (RKKRRQRRR); pVEC—18-amino-acid peptide derived from the murine vascular endothelial cadherin protein; CF—Carboxyfluorescein; TAMRA—tetramethylrhodamine; KAibA—Synthetic CPP with a lysine/α-aminoisobutyric acid/alanine repeat; EDP—Endosome-disrupting peptide.

## 2. Mesoporous Silica Nanoparticles

Mesoporous silica nanoparticles (MSNs) have great potential as a nanomaterial for drug delivery in medicine due to their large surface area, tunable pore size and biosafety [81,82]. One of the main challenges for MSN utilization for nucleic acid delivery used to be the negative charge in hydrophilic conditions. But this obstacle can be successfully overcome because MSNs are rather stable, allowing different chemical modifications for their functionalization [83], e.g., decoration with cationic polymers such as polylysine, polyarginine, polyethyleneimine, etc. (reviewed in [84]). Nucleic acid binds via electrostatic interactions with charged nanoparticles’ surface but the advantage of mesopores per se is not used. Li et al. [85] developed an approach that allows siRNA packaging within the mesopores, thus protecting the nucleic acid, providing its gradual release and delivery into cells. Later, more MSN design variants for “hiding” nucleic acid molecules inside the mesopores were developed (reviewed in [86]).

As for plant cells, MSN-based nanoplatforms have attracted the attention of plant biologists and biotechnologists, but at the moment, there are only few studies (Table 1) demonstrating the successful utilization of MSNs for the delivery of plasmid DNA into plant cells [39,40,41].

Plant cells, as was mentioned earlier, are more challenging objects due to the presence of several mechanical and chemical barriers on the way to the plasma membrane such as the cuticle, cell wall and apoplast. To avoid the most significant plant cell mechanical barrier, the cell wall, researchers often demonstrate the successful penetration of a cargo–carrier complex or cargo into protoplasts [39,40,42]. However, there are several studies demonstrating effective MSN-mediated cargo delivery into intact cells [39,41,42,87,88]. Chang et al. [39] showed in protoplasts and then in *A. thaliana* roots that MSNs functionalized with different organic molecules (3-aminopropyltrimethoxysilane, N-trimethoxysilylpropyl-N,N,N-trimethylammonium chloride, (3-Trihydroxysilyl)propylmethylphosphonate) could be internalized by plant cells and provide intracellular release of pDNA cargo, as was confirmed by monitoring the expression of the encoded fluorescent protein. Moreover, Sun et al. [42] demonstrated that root uptake of ~20 nm MSNs results in their detection in the vascular system and aerial parts of the plants. Thus, there is a great potential for the systemic (throughout the plant) delivery of different cargo using RNA- or DNA-loaded MSNs. MSN/DNA complexes are suggested to penetrate not only the cell but the nucleus, where pDNA release occurs [39]. However, the mechanism underlying these events is unknown and requires further elucidation.

MSNs look like promising nanomaterials for various cargo delivery into plant cells due to their biosafety, biocompatibility and stability. MSNs were shown to be non-toxic in protoplasts in concentrations up to 100 mg/L [40]. However, assessment of their effect on seed germination varies with a low MSN concentration (up to 2 mg/L) and did not affect the germination of lupin, wheat and maize seeds, while higher concentrations (up to 20 mg/L) led to significantly reduced germination [42,43]; on the other hand, Nair et al. (2011) reported that a 50 mg/L MSN concentration did not induce negative effects on rice seed germination [89]. Such a difference could be either species-specific or a consequence of different experimental setups.

Furthermore, MSNs were shown to be applicable to different plant species (*N. tabacum*, *S. lycopersicum*, *A. thaliana*, *Zea mays*) (Table 1) and parts of the plant (protoplasts, suspension culture BY-2, leaves, roots). Despite this potential and the successful application of MSN-based delivery of biologically active cargo, including nucleic acids, to mammalian cells, only a handful of studies demonstrate the application of these tools to plants. Mainly, MSNs are regarded as carriers for the biolistic transformation or direct injection for transient expression and the obtainment of stably transformed plants [40,44,90,91,92]. Biolistic MSN-mediated cargo delivery was utilized not only for plasmid DNA but for biologically active proteins as well: Martin-Ortigosa et al. [91] made a successful attempt to use gold-plated MSNs for Cre recombinase delivery into *Zea mays* cells harboring loxP sites flanking a selection gene and a reporter gene. The calli obtained from the treated material contained cells with the edited genome, confirming that Cre–MSN particles were correctly delivered and functionally active Cre released from the complex. Thus, this application of MSNs could also be regarded as a very prospective tool for active enzyme delivery into plant cells, especially if an alternative approach to the biolistic method were developed.

Silica is a natural compound that is in contact with plants; moreover, it is biodegradable and is believed to be non-toxic. MSN/DNA complexes could be used as an alternative for biolistic transformation or protoplast transfection for obtaining transgenic plants (for those species that cannot be transformed using Agrobacterium). However, the final intracellular destination of the cargo should be taken into account because MSNs were reported to be detected mainly in the chloroplasts [40,42] or in the nucleus [44] of treated cells. Despite the lack of studies on MSN-mediated RNA delivery in plants, the potential for this nanocarrier utilization for RNA packaging has been demonstrated [85]. Finally, additional biosafety and toxicity analysis of each particular MSN’s variant preparation and functionalization should be performed, as well as research on the intraplant distribution and long-term effects, before this technology could be transferred to the field for agricultural purposes.

## 3. Layered Double Hydroxides

Layered double hydroxides (LDHs), or so-called anionic nanoclays or hydrotalcite-like systems, are based on the initially discovered natural magnesium–aluminum hydroxyl carbonate–hydrotalcite: [Mg_6_Al_2_(OH)_16_](CO_3_)·4(H_2_O). They are characterized by good biocompatibility and high chemical stability [93] and are gaining popularity as platforms for the delivery of different compounds into living cells. Due to positive charge, they successfully bind nucleic acid, protecting it from different environmental challenges such as UV light, rain and enzymatic and chemical damage [31,47,48]. LDH sheets are stable at a pH higher than 6, while at an acidic pH, characteristic of plant cells and apoplasts, the release of cargo is observed [31]. dsRNA incorporated into LDH sheets could be detected on the sprayed leaf surface up to 20 days after application [23], allowing a gradual and controlled dsRNA release rate, followed by penetration into the leaf tissues and providing a prolonged silencing effect. In another study, *Solanum lycopersicum* plants were treated with dsRNA for genes essential for *Fusarium oxysporum* effective plant colonization. The LDH/dsRNA complexes of 30–90 nm were applied to the tomato plants in three ways: foliar spraying, petiole adsorption and dipping the roots into solution. It was demonstrated that all three approaches were efficient: they provided increased resistance to *F. oxysporum* and prevented the development of severe symptoms such as crown and root rot for up to 60 days on the monitored plants [48]. Likely, LDH/dsRNA complexes do not enter the plant cells, remaining in the apoplastic space and xylem and not being processed into siRNA in planta, when applied via petiole adsorption. However, the most prominent protective effect was obtained when LDH/dsRNA-containing solution was sprayed onto the leaves. These results allow authors to suggest that in the case of foliar spraying, dsRNA enters the plant cells where it is processed in addition to the dsRNA that remained in the apoplast, thus inducing the silencing of the essential *F. oxysporum* genes both by fungus- and plant-generated siRNAs. These results indicate that LDH/dsRNA tomato plant pre-treatment efficiently protected them from *F. oxysporum* infection via RNAi.

Besides dsRNA delivery using LDHs as a carrier, another approach was developed for fighting tomato yellow leaf curl virus (TYLCV) infection: a mixture of pDNA encoding three artificial microRNA against TYLCV was loaded into LDH and applied to *S. lycopersicum* or *N. benthamiana* plants via spraying. It was shown that pre-treatment with pDNA/LDH made plants more resistant to TYLCV infection [31].

LDH internalization was under question for a long time. It was considered that LDHs were likely not to be internalized into plant tissues; they would stay on the surface and provide dsRNA protection together with gradual release and further internalization of dsRNA. But the question of whether LDH nanoparticles could enter the plant cells and the apoplast, followed by distribution via the vasculature, was answered only recently. Earlier, it was demonstrated that delaminated LDH-lactate nanosheets (30 nm in diameter and 0.5 to 2 nm thick, monolayer or bilayer) were able to enter BY-2 tobacco cells and *Arabidopsis thaliana* roots. Additionally, it was shown that LDH-lactate/nucleic acid nanosheets enter the cell via a non-endocytic pathway because the typical inhibitors of endocytosis do not interfere with this process [45]. Further, Prof. Xu’s group showed that the size threshold of particle size penetration is about 50 nm, so studied 50 nm LDH/dsRNA nanoparticles could enter cells of the developing *S. lycopesicum* pollen [46]. To reveal whether such nanoparticles could enter leaf cells, the same research group analyzed a 40 nm LDH/dsRNA nanoparticle distribution after the infiltration of *N. benthamiana* leaves. They showed that LDH/cargo complexes entered the apoplast, were internalized by the cells delivering their cargo and could be detected in the vasculature of the plant [47]. Moreover, it was demonstrated that LDH-incorporated cargo (siRNA, Cy5-labeled DNA) entered the chloroplasts due to the LDH’s positive charge and the electronegative nature of the inner surface of the plant membranes. This difference in charge likely facilitates the penetration of LDH-protected cargo into the cells and cellular compartments. Notably, even negatively charged cargo could be delivered into cells in a complex with LDH as the carrier isolates the cargo from the environment. This was confirmed using pH-sensitive FITC dye, which almost completely lost the ability to fluoresce inside the apoplast and cytoplasm of the plant cell, where the pH is about 5–6. But, incorporated into LDH, it remains fluorescent after cellular uptake [47].

Thus, LDH-based materials were confirmed to be very promising nanocarriers for DNA and RNA delivery into plant cells, and are characterized by biosafety, being environment-friendly. Atmospheric CO_2_ and water can slowly break down LDHs into biocompatible components. One of the main advantages of this nanoplatform is the efficient protection of such a vulnerable cargo as DNA and RNA, keeping them safe on the leaf surface. Also, it mediates gradual cargo release, providing a prolonged effect. LDH-based nanomaterials were tested on several species. But studies on the assessment of interspecies leaf surface differences’ impact on the protection efficiency have not been performed yet; thus, it can be only suggested that LDHs are universal for various plant species. Moreover, as for most of the nanomaterials, the mechanism of the cargo and nanoparticle internalization and intraplant distribution is still understudied and should be elucidated.

## 4. Carbon-Based Nanoplatforms

There is a great variety of nanomaterials based on carbon. Carbon nanomaterials could be classified into several categories such as nanotubes, fullerenes, nanoparticles, nanohorns, nanobeads, dots, nanofibers and nanodiamonds [94]. Here, we discuss the advantages and limitations of only two groups—carbon dots (CDs) and single-walled carbon nanotubes (SWNTs)—as the most perspective carbon-based nanoplatforms for the delivery of nucleic acids into plant cells.

### 4.1. Carbon Dots

Carbon dots (CDs) are carbon-based nanomaterials that are characterized by excellent biocompatibility, stability, low toxicity, water solubility and small size below 20 nm, which contributes to rather effective cellular uptake, as was shown in studies on animal [95,96] and plant cells [97,98,99]. Moreover, now, they are regarded as a non-toxic alternative to heavy-metal-based quantum dots, the cellular uptake of which has been studied in plants [100]. CDs can be obtained either from graphene as a precursor or from biomass and different organic compounds such as amino acids, sugars, etc. [101]. In addition to the abovementioned advantages of CDs, they possess the ability to emit fluorescence with a high quantum yield and resistance to photobleaching as their intrinsic properties [102,103,104]. CDs can be passivated with different molecules, for example, polyethylenimine (PEI), to acquire a positive charge for further DNA or RNA cargo binding. However, this leads to an increase in toxicity [105] and should be taken into account when working with living systems. Studies on the uptake of water-soluble CDs in wheat show that CDs are absorbed by plants and such treatment leads to an increase in root and shoot growth. Thus, authors claim that CDs are not toxic and suggest that they could be used as a booster to increase crop productivity [97]. Results demonstrating a positive effect on plant growth and metabolism have also been obtained for mung beans [106,107], and lettuce [108]. Tobacco plants’ treatment with CDs led to increased efficiency in photosynthesis [109]. On the other hand, a study by Chen et al. [98] demonstrated the dose-dependent phytotoxicity of CDs applied to maize seedlings and plants: the authors observed membrane damage, oxidative stress and the activation of antioxidant enzymes in response to a high concentration of CDs. Thus, the effects of carbon-based nanomaterials, including CDs, on plants is far from well studied and is still to be elucidated [94]. However, CDs were demonstrated to be efficiently internalized by plant cells and transferred via vascular systems throughout the plant in different species. Therefore, CDs could be regarded as nanoplatforms for the delivery of biologically active cargo into plant cells. At present, there are only few successful examples of using CDs as carriers for the delivery of nucleic acids (Table 1) [29,49,50] as this nanomaterial only recently started to be regarded as a tool for this application despite there being numerous studies on plant and animal cells [110]. Schwartz et al. [29] demonstrated the suitability of PEI-functionalized CDs for the efficient delivery of siRNAs into the cells of *N. benthamiana* and *S. lycopersicum* leaves via spraying. Moreover, systemic silencing was observed both of the transgenes (*GFP*) and of endogenous magnesium-chelatase-encoding genes. In addition, PEI–CDs were demonstrated to be applicable to nucleic acids delivery in other dicot and monocot species—*O. sativa*, *T. aestivum*, *Phaseolus radiates*—as was shown by Wang et al. for pDNA [49] and by Delgado-Martín et al. for dsRNA delivery in *Cucumis sativus* seedlings [50].

To summarize, functionalized CDs prove to be promising nanocarriers with additional beneficial properties (e.g., the ability to stimulate photosynthesis, root and shoot growth and plant productivity in general). They are suitable for species from different taxonomic groups. However, one of the main limitations of CDs utilization is their dose-dependent toxicity, which should be thoroughly investigated. Moreover, the biosafety issues and potential long-term effects of CDs accumulation in plant materials, soil and water are to be the subject of further studies.

### 4.2. Carbon Nanotubes

Single-walled carbon nanotubes (SWNTs) are low-dimensional cylindrical tubule structures made from graphite. This nanomaterial possesses unique physical and chemical properties that allow using it to shuttle inside living cells various molecular cargos, including drugs, proteins, peptides and nucleic acids [111,112]. SWNTs have a needle-like structure with a diameter 1–3 nm and a length of about few micrometers; they can be functionalized with different additional groups [54,113]. SWNTs feature a unique combination of rigidity, strength and elasticity compared with other fibrous materials. Moreover, SWNTs are fluorescent in a near-infrared wavelength, allowing their imaging and tracking in biological systems [114]. Another beneficial feature of SWNTs is their antifungal and antibacterial effect [113]. The nanotubes are relatively biocompatible but their cytotoxicity depends on the linked functional groups and concentration, as shown in mammalian cell cultures [115,116,117,118,119]. As for plant cytotoxicity studies, leaf infiltration with pristine SWNTs leads to a mild stress response similar to the reaction induced by mock infiltration; however, PEI-decorated SWNTs in comparison with pristine SWNT treatment result in more significant transcriptional reprogramming that affects the stress response; high concentrations of SWNTs could suppress photosynthesis and cause cell death [120,121]. The cytotoxicity of carbon nanotubes could be modified via their surface functionalization, e.g., SWNT conjugation with arginine [116] or the utilization of different PEI variants (low-molecular-weight linear PEI, hydrophobically modified branched PEI and high-molecular-weight branched PEI, etc.) [120,122], leading to a reduction in their cytotoxicity. On the other hand, different types of carbon nanotubes in concentrations 10-100 µg/L with the optimum ~40 µg/L were shown to have beneficial effects on plant water uptake and seed germination. Such growth stimulation in the presence of carbon nanotubes was attributed to the increase in nutrient uptake due to improved water delivery. However, higher concentrations led to the contrary effect, reflecting the concentration-dependent cytotoxicity of this material. For example, exposure of *A. thaliana* and rice protoplasts to SWCNTs led to oxidative stress and programmed cell death [123]. All these observations underline the importance of the correct dosage to be used for plant treatments (reviewed in [94,113]).

SWNTs could efficiently protect nucleic acids from extra- and intracellular degradation and enzymatic cleavage and prevent their interaction with nucleic acid binding proteins [53,124,125]. Due to these features, SWNTs have successfully been used as a nanocarrier for nucleic acid delivery in plant cells across the cell wall and membrane (Table 1) [28,52,53,55,56,126]. There are several approaches to obtaining SWNT complexes with nucleic acids: (i) SWNT functionalization using PEI or another polycationic substance (e.g., chitosan, arginine-enriched peptide) to gain a positive charge and to bind DNA electrostatically [28,52,56,126]; (ii) SWNTs are initially coated with sodium dodecyl sulfate that desorbs from the SWNTs’ surface during dialysis and is replaced with DNA, that adsorbs onto the surface of the SWNTs via π–π stacking interactions [54].

Liu et al. showed that an SWNT complex with single-stranded (ss) DNA can penetrate the cell wall: SWNT/ssDNA–FITC conjugates were quickly delivered into the intracellular space of *N. tabacum* BY-2 cells and observed using fluorescence microscopy [52]. The efficiency of delivery was high: intracellular fluorescence was observed for more than 80% of the cells that were incubated with the SWNT/ssDNA complex. Of note, the control SWNT/FITC complexes were localized to the vacuoles, while the SWNT/ssDNA–FITC fluorescence was distributed in the cytoplasm [52]. SWNT-mediated pDNA delivery in the cells of *N. benthamiana*, *Eruca sativa* (arugula), *Triticum aestivum* (wheat) and *Gossypium hirsutum* (cotton) leaves and arugula protoplasts was demonstrated by Demirer et al. [53,54]. To obtain SWNT/pDNA complexes, carboxylated SWNTs were covalently modified with polycationic PEI to gain a positive charge for the electrostatic binding of negatively charged pDNA. PEI–SWNT/pDNA nanoparticles infiltrated into the leaves of the abovementioned plants and internalization of the pDNA was confirmed by the detection of the encoded gene expression either using fluorescence microscopy or qRT-PCR [54]. Dunbar et al. performed the delivery of different pDNA into *O. sativa* leaves and embryos using PEI–SWNT as a carrier. After infiltration of the rice leaves and seeds with PEI–SWNT/pDNA, transient expression of either GFP, YFP or GUS was detected [28]. Another variant of SWNT functionalization was performed by Golestanipour et al. [126]: they used SWNTs conjugated with arginine to obtain a positively charged nanocarrier. SWNT functionalization with Arg reduces cytotoxicity, as was demonstrated earlier [116]. Arg–SWNT could successfully deliver GFP-expressing plasmids into tobacco root cells as was confirmed using fluorescence microscopy and Western blot analysis [126].

A SWNT-based approach is applicable to the efficient delivery of siRNA into plant cells for RNAi induction, as was shown in *N. benthamiana* plants containing the *GFP* transgene [124]. Silencing was induced within 6 h after infiltration. Cy3-labeled siRNA/SWNT complexes were observed inside the leaf cells and in the extracellular space. *GFP* silencing was prolonged by the reinfiltration of another siRNA/SWNT dose 5 days after the first infiltration. Adsorption on the SWNTs provides protection of the siRNA cargo from nuclease degradation for up to 24 h, whereas free siRNA is degraded in a few hours. Of note, in this experimental setup, two types of RNA/SWNT complexes were mixed: each contained one RNA strand—either sense or antisense. The sense and antisense strands of siRNA were noncovalently adsorbed on the SWNTs via π–π stacking of the RNA nitrogen bases with the π bonds of sp^2^-hybridized carbon in the SWNTs. The resulting double-stranded siRNA formed inside the cells. This approach appeared to be effective for the silencing of endogenous gene *ROQ1* [124]. Demirer et al. [127] also developed a detailed protocol of siRNA molecule delivery into plant cells using SWNTs.

SWNT-based complexes allow the delivery of nucleic acids (pDNA) not only into plant cells but into particular organelles: chloroplasts [56] and mitochondria [55]. Kwak et al. [56] developed a nanocarrier based on SWNTs decorated with deacetylated chitosan. This nanoplatform was used to deliver pDNA into the chloroplasts of different plant species including *E. sativa*, *Nasturtium officinale*, *N. tabacum* and *Spinacia oleracea*. But, first, this approach was shown to be effective for *A. thaliana* isolated mesophyll protoplasts. Several types of chitosan–SWNT conjugates were tested: non-covalent SWNTs wrapped with chitosan or PEGylated chitosan and SWNTs with covalently bound chitosan. Plasmid DNA was complexed with a positively charged chitosan–SWNT carrier via electrostatic interactions. The chitosan–SWNT/pDNA nanoparticles were shown to efficiently penetrate the *A. thaliana* protoplasts and enter the chloroplasts. pDNA was released from the complexes due to the pH shift from slightly acidic, characteristic of the cytoplasm, to basic inside the chloroplast stroma. Moreover, no nuclear targeting and pDNA expression were observed; thus, a chloroplast-specific release was confirmed. Along with chloroplast targeting, Law et al. [55] developed an approach to SWNT-based pDNA delivery into mitochondria. SWNTs coated with a cross-linked polymethacrylate maleimide polymer network (SWNT–PM) were decorated additionally with Cyt peptide (MLSLRQSIRFFKC), mediating mitochondria targeting, and KH9 peptide for cell penetration (KHKHKHKHKHKHKHKHKHC). This combination of two different nanoplatforms (SWNT and CPP) allowed the researchers to obtain a near 30-fold increase in reporter gene delivery and expression without toxicity effects compared to nanoparticles based on the same CPPs only tested in previous studies [80]. The SWNT/peptide hybrid nanocarrier (SWNT–PM–CytKH9) was shown to successfully mediate pDNA delivery into the mitochondria of *A. thaliana* seedlings and roots via vacuum infiltration. The luciferase and GFP expression from the delivered pDNA was detected in the cytoplasm and mitochondria of the treated seedlings. Of note, no significant expression was detected when SWNT–PM–KH9/pDNA complexes were applied despite the SWNT–PM–KH9 carrier having the highest pDNA binding capability in the binding assays. The authors explain this with the too tight interaction between the KH9 and pDNA leading to the inefficient release of nucleic acid from the complex in the mitochondria. Therefore, the optimal variant of the nanocarrier based on CPPs and SWNTs for the delivery of pDNA into the mitochondria was SWNT–PM–CytKH9, which provided efficient penetration and targeting [55].

Despite limited data on SWNTs as a carrier for nucleic acid delivery into plant cells, the “proof-of-concept” studies demonstrating successful pDNA and siRNA delivery open up a perspective on the utilization of SWNT-based nanoparticles for the induction of RNA interference by treating plants with dsRNA/SWNT or siRNA/SWNT complexes for crop protection from viral infection and the modulation of internal gene expression. However, the limitations for SWNTs utilization are similar to those for CDs: toxicity and lack of information on their long-term effects. The fate of SWNTs within plants and in the environment is an important issue to be addressed in future studies.

## 5. Chitosan-Based Nanocarriers

Chitosan is a linear β-1,4-d-glucosamin polymer which is obtained in several steps from natural or synthetic chitin via deacetylation [128]. Industrially made chitosan is not a homogenic substance: it consists of polymers of different lengths and molecular weights with various degree of acetylation. Thus, for the preparation of nanoparticles to utilize them for cargo delivery into living cells, it should be taken into account that chitosan’s physical and chemical properties such as solubility, viscosity, etc. depend on its molecular weight and homogeneity [129,130]. Chitosan, due to its relatively low price, availability, biocompatibility and biosafety, has numerous applications, including in the biomedical [131,132], agricultural [130,133] and food industries [134], and others [129].

Chitosan -NH_2_ groups could be protonated into -NH_3_^+^ in slightly acidic conditions, giving the whole molecule positive charge. This feature allows it use as a nanocarrier for nucleic acid delivery. In addition to studies on mammalian cells [135,136,137], there is evidence of the successful use of chitosan/dsRNA complexes to induce the silencing of different genes in insects [138,139], including plant-feeding pests [140,141,142]. Chitosan-based nanoparticles protect dsRNA (or DNA) from degradation and unfavorable environments.

As far as plants are concerned, only a few examples of chitosan-based formulations for nucleic acid delivery into plant cells have been described (Table 1). The majority of studies on plants is devoted to chitosan’s application as a substance against pathogenic bacteria, fungi or pests, as well as a growth stimulator. Chitosan-based nanoparticles could provide gradual and controlled release of cargo as was demonstrated for various nutrients, fertilizers, pesticides and other compounds used in agriculture [143,144,145]. Foliar applications of chitosan and chitosan-based nanoparticles were demonstrated to have antibacterial [146], antifungal [147,148] and antiviral [149,150] activity. Moreover, chitosan increases plant productivity due to the activation of enhanced stress resistance and metabolic stimulation (as reviewed in [24,130]). In addition to the numerous beneficial properties of chitosan, it was demonstrated on armyworm that it helps dsRNA to escape endosomes, which leads to more efficient RNAi [140]. Endosomal escape is realized via the so-called “proton sponge effect”: chitosan’s amino groups are protonated in the endosome’s acidic conditions, which leads to the sequestering of water and chloride ions from the endoplasm, resulting in endosome disruption. But for plant cells, no similar studies have been performed yet. However, as the general route of endocytosis is similar throughout eukaryotes, these results could potentially be extrapolated to plant cells.

Despite the numerous beneficial properties of chitosan and a great number of studies on chitosan applications, only a few examples of chitosan-mediated delivery of nucleic acids into plant cells have been described currently [51,57,59,60,61,62].

Nanoparticles from tripolyphosphate (TPP)-crosslinked chitosan were demonstrated by Makhotenko et al. [57] to serve as a carrier for the delivery of a protein (bovine serum albumin) and a small RNA (tRNA) into *N. benthamiana* cells via syringe infiltration. Moreover, the same TPP–chitosan nanoparticles were demonstrated to efficiently deliver a ribonucleoprotein complex containing Cas9 endonuclease and guide RNA into the cells of the potato apical meristem via vacuum infiltration, which was confirmed via the sequencing of edited coilin [60] and phytoene desaturase (PDS) [59] genes in regenerated cell lines. Zhang et al. [61] used chitosan quaternary ammonium salt to obtain nanoparticles with incorporated pDNA. The chitosan/pDNA nanocomplexes were infiltrated into *N. benthamiana* leaves. The pDNA-encoded gene *NbMLP28* that was earlier shown to increase resistance to potato virus Y (PVY) [30] was used to assess the efficiency of delivery. Its expression was detected either using fluorescence of its fusion with RFP or in a functional test, in which increased resistance to phytoviruses was demonstrated in plants treated with chitosan/pDNA complexes. However, in the abovementioned studies, nanoparticle application was performed via infiltration. The ability of chitosan-based complexes to penetrate into plant cells after topical application was recently demonstrated by Xu et al. who used different approaches, including leaf spraying and root soaking for the delivery of dsRNA into the cells of *Solanaceae* plants (tobacco, pepper, tomato), resulting in the induction of antiviral protection against PVY [51] and tobacco mosaic virus (TMV) [62]. In the described experimental setup, chitosan/dsRNA nanoparticles were applied to *N. benthamiana* plants followed by PVY inoculation. The efficiency of infection was assessed by measuring the level of PVY RNA in the non-treated new leaves 3 weeks after inoculation. The most powerful PVY suppression effect was observed at the 21^st^ day post inoculation in plants pre-treated with chitosan/dsRNA via root soaking or leaf spraying in contrast to infiltration [51]. Moreover, field experiments on tobacco, pepper and tomato were performed using spraying application of chitosan/dsRNA. It was shown that the treatment of plants with chitosan/dsRNA complexes was safe and effectively lowered the disease index [51]. Another study from the same research group describes the anti-TMV effect of dsRNA (against the replicase gene) delivered into tobacco plants in a complex with chitosan [62]. The focus of that research was on the prevention of TMV seed-mediated transmission. The chitosan/dsRNA complexes were applied via leaf infiltration or spraying, root soaking and pollen incubation. The latter was the most effective route of dsRNA delivery for the prevention of seed contamination with TMV. As for foliar application, infiltration was shown to be more efficient than spraying [62] in contrast to the results obtained for PVY [51]. Nevertheless, the best antiviral effect both in the leaves and in pollen (and seeds) was obtained after root soaking, which allowed systemic distribution of dsRNA throughout the plant. According to the authors’ assumptions, the chitosan/dsRNA nanoparticles adhered to the cell wall of the root cells and were internalized by the cell due to the concentration difference; then, through the vascular tissues, they were delivered to the upper leaves. Induction of RNA interference was also confirmed via the sequencing of the small RNA: siRNA corresponding to the TMV replicase gene was detected, indicating the efficient activation of an antiviral response [62].

However, there are some limitations in relation to chitosan nanoparticle utilization. Mostly, they are connected with the nanoparticle preparation and the quality of the initial material: the properties of chitosan-based nanoparticles are highly dependent on the homogeneity of the initial polymer and its acetylation degree. Each preparation of nanoparticles should pass quality control and be thoroughly characterized. Moreover, the mobility and distribution of chitosan-based nanocarriers throughout the plant is not yet fully understood, so potential adverse effects cannot be excluded, as the main data on biosafety and biocompatibility have been obtained in animal systems. Nevertheless, despite only a few studies in plants being available at present, chitosan-based nanoparticles have proved to be applicable as a carrier for nucleic acid delivery into plant cells. They allow penetration through mechanical barriers (cuticle and cell wall) and mediate endosomal escape. Taking into account the other beneficial properties of chitosan, such as its antifungal, antibacterial and antiviral activity as well as plant growth stimulation, this material has great potential as a nanocarrier for dsRNA and DNA delivery and obtaining multiple protective effects on crop plants.

## 6. Cell-Penetrating Peptides

The first discovered cell-penetrating peptides were of viral origin: a domain of human immunodeficiency virus (HIV) trans-activator of transcription (Tat) protein was shown to be responsible for cellular uptake and penetrating the membrane [151,152,153]. The Tat protein transduction domain contains a positively charged amino acid sequence YGRKKRRQRRR that could mediate cell entrance of the target protein [154]. Later, based on this sequence, numerous CPPs were found in natural sources or were developed artificially. The CPPsite 2.0 database (https://webs.iiitd.edu.in/raghava/cppsite/index.html, accessed on 15 May 2023) of experimentally validated CPPs [155] contains at the moment about 1700 unique peptides of different natures. Their common features are a length less than or about 30 amino acid residues and a positive charge. Also, some peptides have amphipathic properties. Usually, such peptides are rich in arginine, lysine and histidine residues and could have additional functional parts [156]. CPPs could facilitate penetration through the plasma membrane; thus, they have been successfully exploited for different cargo delivery into animal cells [157,158]. However, their utilization for plant cells is much more challenging because of additional mechanical barriers. A nanocarrier/cargo complex should be stable in an extracellular environment, be able to penetrate the cuticle and cell wall, translocate through the membrane and, finally, have the ability to escape the endosome and release the cargo into the desired cellular compartment. CPPs possess the majority of these required features, allowing cell penetration and endosomal escape.

There are several examples of natural-protein-originating CPPs tested in plant systems. In addition to the Tat-derived peptide [64], an 18-aa pVEC peptide from the murine vascular endothelial cadherin protein [159] was shown to penetrate into wheat mesophyll protoplasts and other plant tissues [65,66]. Also, a 27-aa synthetic peptide transportan [160] that contains a fragment of the natural neuropeptide galanin and hydrophobic toxin mastoparan was tested in the same experimental system, showing similar results [65,66]. However, in these studies, no cargo was complexed with the peptides, and their cellular uptake was confirmed via the microscopy of fluorescent dye linked to them.

As was mentioned above, the first discovered CPP was of viral origin. Later, several more sequences that have properties of CPPs were detected in the proteins of other animal viruses [161,162,163]. As for plant viruses, there is an example of a viral protein showing the features of CPPs [63]. Brome mosaic virus (BMV) capsid protein contains an N-terminal arginine-rich sequence that has the ability to bind RNA. BMV particles were demonstrated to enter barley protoplasts. Moreover, it was demonstrated that capsid protein N-terminal residues 9–22 are responsible for this internalization, as they were able to mediate GFP entrance into protoplasts as well. Labeled with fluorescent dye, the BMV CPP was taken up by *Arabidopsis* and barley roots and passed through the barley mesophyll cell wall and plasma membrane, entering the mesophyll cells. Additionally, it was shown that the BMV CPP, possessing an RNA-binding capacity, could mediate the intracellular delivery of both BMV RNA and in vitro-synthesized dsRNA into barley protoplasts. Capsid proteins from other viruses from different taxonomic groups—barley yellow dwarf virus (BYDV), tobacco curly shoot virus (TCSV) and bean yellow dwarf virus (BeYDV)—were demonstrated to contain similar CPP sequences that were able to enter *Arabidopsis* and barley roots and barley leaf mesophyll cells [63]. Thus, such natural CPPs of viral origin could be regarded as potential instruments for dsRNA delivery into plant cells.

However, the most successful results on the utilization of CPPs as carriers for nucleic acids were obtained with different positively charged synthetic peptides or CPPs decorated with additional organic groups. During the last decade, numerous studies demonstrating the great potential of CPPs and their ability to deliver various cargo—plasmid DNA [27], siRNA and dsRNA [26,75] and protein [72,164]—into plant cells were performed by Dr. Numata’s research group (Table 1). Screening of a 55 CPP library containing amphipathic, hydrophobic and cationic peptides allowed the authors to identify those that were efficiently internalized into BY-2 cells or the cells of *N. benthamiana*, *A. thaliana*, *S. lycopersicum*, poplar leaves, and *Oryza sativa* callus. Ultimately, 8 of the 55 CPPs were chosen as the most effective for all tested plants [70]. Among those selected peptides were transportan, arginine-rich peptide R12, three amphipathic peptides and one hydrophobic peptide. In other studies, peptides consisting of a BP100 CPP (KKLFKKILKYL) [165] and lysine/histidine copolymer (KH)_9_ were demonstrated to be effective for mediating pDNA, dsRNA or protein delivery into plant cells [26,27,72,75]. First, these peptides were tested using an infiltration approach and the treatment of BY-2 cells. Further, a very important upgrade was performed when topical application via the spraying technique was shown to be also effective for the delivery of either dsRNA or pDNA in a complex with CPPs [75].

In addition to the efficient intracellular delivery of the cargo, another important aspect should be taken into account: cargo endosomal escape inside the cell. Despite multiple studies on the CPP/cargo internalization mechanisms having been performed, they have still not been fully clarified. For some peptides, it was demonstrated that they enter the cell endocytosis independently, but others are sensitive to the inhibitors of endocytosis [166]. Endocytic vesicle escape probably is achieved due to the pH-buffering properties of the carrier peptide (for example, histidine residues). But the efficiency of this and its correlation with the peptide composition is still to be elucidated. To overcome this obstacle and suggest a reliable approach to bypassing the vacuolar degradation of the cargo, Miyamoto et al. [73,74] developed a novel type of nanocarrier formulation: a poly-Lys or/and Lys–His co-polymer conjugated with maleimide via tetra ethylene glycol was used as a platform that formed micelles with pDNA via electrostatic interactions. Due to the maleimide groups exposed on the surface, these micelles could be further decorated with additional functional peptides: CPPs and organelle-targeting or endosome-disrupting peptides (EDPs). Several combinations of CPPs and EDPs were tested and the approach was demonstrated to be effective for the cellular and nuclear delivery of pDNA into plant cells using vacuum infiltration [73]. EDPs were confirmed to significantly elevate the efficiency of this process, allowing pDNA-containing micelles to escape from the endosome in *A. thaliana* seedling tissues [74].

When all the chemical and mechanical barriers are overcome, another aspect arises: the release of the cargo from the nanocarrier-containing complex if the whole nanoparticle was internalized. For efficient and controlled intracellular cargo release, Chuagh and Numata [71] designed a stimulus-responsive peptide BPCH7 based on the BP100 CPP conjugated with CH7 peptide (HHCRGHTVHSHHHCIR). CH7 peptide represents a reducible pDNA-binding domain that, after cellular entrance, releases pDNA in the presence of reduced glutathione (GSH). Because this peptide contains an internal disulfide bond, its reduction by GSH leads to conformational changes. The cyclic properties of BPCH7 are likely another factor that allows the early endosome escape of the complex. The same peptide was shown to efficiently deliver protein cargo (yellow fluorescent protein citrine) in the cells of *O. sativa* callus [72] after infiltration.

One of the directions of CPP application is the organelle-specific delivery of cargo. As was mentioned above, good results were obtained in transferring pDNA to the nucleus, followed by the successful expression of the reporter gene. This type of cargo is easily transferred to the nucleus because the utilized CPPs usually contain residues of Arg and Lys, making the sequence similar to the nuclear localization signal that is recognized by the eukaryotic cellular factors of nuclear import.

Utilization of chloroplast transit peptides [76,77,78,79] or mitochondria-targeting [76,80,167] signal peptides in combination with CPPs was demonstrated to be an efficient tool for the delivery of different cargo into the corresponding plant cell organelles. Two main approaches are used: an organelle-targeting peptide is fused to a CPP [78,79] or a complex is formed first of the cargo and organelle-targeting peptide, followed by the further nanoparticle decoration with a CPP [77]. This approach could be used to obtain plants with stably transformed chloroplasts without using such special equipment as a gene gun. Odahara et al. have demonstrated effective peptide-based chloroplast transformation for three species: tobacco, rice and kenaf [79]. As for mitochondria transformation, a pioneer successful study on a whole plant (*A. thaliana*) without using a gene gun or isolating mitochondria was conducted by Dr. Chuah [80]. The authors obtained complexes of pDNA with the originally designed peptide consisting of a mitochondria-targeting peptide (MTP) fused with a Lys/His-rich CPP (KH)_9_ or BP100-(KH)_9_. Further, this approach was applied to mitochondrial genome transformation via homologous recombination [76] and demonstrated to be suitable not only for *A. thaliana* but for tobacco as well.

Summarizing the results obtained utilizing different types and combinations of peptides, based on the CPPs, we could conclude that they are very powerful tools for the delivery of various cargo into intact plant cells. The main advantages of this approach, besides biocompatibility, low toxicity and other essential features, is applicability both to standard model plants and to non-model plants, including important crops, among which are dicot and monocot species. Moreover, such peptides represent a tool for mitochondrial genome transformation as well as for the transformation of chloroplasts. The success was achieved using different types of plant treatment: from co-incubation with the solution of peptide/cargo complexes to infiltration and topical application via spraying, which looks a very promising technique for scaling up and transferring this approach to the field. Future studies should aim to increase the efficiency of CPP-mediated delivery, as this is the main limitation of this nanocarrier.

## 7. Concluding Remarks

RNAi is a valuable tool in plant disease and pest management. dsRNA/siRNA topical application for the induction of RNAi is undeniably a very prospective technology that was demonstrated in laboratory studies to have great potential. Moreover, first attempts to transfer this technology to field conditions have been made, showing the suitability of this approach for sustainable agriculture. However, multiple hurdles are still to be overcome: one of the most significant of them is the intracellular delivery of nucleic acids. Recent progress in the development of nanomaterials has led to the enhanced efficiency of the delivery. Moreover, besides its mere penetration into the cell, the problem of nucleic acid’s protection from abiotic factors and the plant’s intercellular and intracellular environment has been addressed. Such nanomaterials as nanoclays provide increased stability of the RNA and its gradual release from the complex. The intracellular fate of the delivered nucleic acid also attracts researchers’ attention, as it is an important step at which the efficiency of the whole process could be increased. Here, questions of the endosomal escape and timely cargo release from the complex with the nanocarrier are raised (Figure 1). Chitosan, for example, has an intrinsic property that allows it to escape from endosomes, while other carriers could be supplemented with functional groups, as was tested with CPPs. Moreover, nanoparticles that can deliver nucleic acids to different compartments have been developed. However, the mechanisms of nanoparticles’ plant and cellular uptake are still understudied, as well as the factors defining systemic RNAi spread and transitivity. Despite numerous “proof-of-concept” studies where successful RNA delivery was confirmed and RNAi induction demonstrated, the particular delivery technique and efficiency of this process is questionable in terms of the scalability aspect. For example, leaf infiltration or petiole absorption could hardly be regarded as approaches for in-field application. Nevertheless, several nanoplatforms discussed here, are shown to be suitable for efficient RNA delivery via foliar spraying without abrasion or pressure (Figure 2). The first success with spray-induced gene silencing became a breakthrough that gives us an opportunity to scale up plant treatment without obtaining transgenic plants or performing genome editing, instead just spraying plants with a solution of nanocarrier/dsRNA complexes to obtain local or even systemic silencing in the field. Foliar spraying is likely the only type of treatment that could be scaled up. Another application method that could be used in agriculture is root soaking; however, it is not suitable for plants grown in soil. Nevertheless, for such closed systems as greenhouse hydroponic cultivation, it could be advantageous. Root uptake of the naked dsRNA does not lead to RNAi induction because such dsRNA cannot enter the cells, remaining in the apoplast. But nanocarriers facilitate RNA penetration from the apoplast into the cell, followed by dsRNA processing and the activation of RNAi.

To summarize, the utilization of nanocarriers for nucleic acid delivery into plant cells greatly facilitates this process and significantly increases its efficiency. Moreover, it offers an opportunity to target RNA/DNA to the desirable compartment. Further studies should aim to understand the fate of the nanocarrier both inside the plant and in the environment, and investigate the potential off-target action of dsRNA/siRNA because one of the important aspects that separates us from transferring the technology to in-field applications is biosafety issues.

## Data Availability

Not applicable.

## Abbreviations

APTES	Aminopropyl triethoxysilane
APTMS	3-aminopropyltrimethoxysilane
BeYDV	Bean yellow dwarf virus
BMV	Brome mosaic virus
BYDV	Barley yellow dwarf virus
CD	Carbon dot
CF	Carboxyfluorescein
CMV	Cucumber mosaic virus
CPP	Cell-penetrating peptide
GUS	β-glucuronidase
EDP	Endosome-disrupting peptide
FITC	Fluorescein isothiocyanate
HACC	N-2-hydroxypropyl trimethyl ammonium chloride chitosan
KAibA	synthetic CPP with a lysine/α-aminoisobutyric acid/alanine repeat
LDH	Layered double hydroxide
MSN	Mesoporous silica nanoparticles
MTP	Mitochondria-targeting peptide
pDNA	Plasmid DNA
PEI	Polyethylenimine
PMMoV	Pepper mild mottle virus
pVEC	18-amino-acid peptide derived from the murine vascular endothelial cadherin protein
PVY	Potato virus Y
RITC	Rhodamine B isothiocyanate
SWNT	Single-walled carbon nanotube
TAMRA	Tetramethylrhodamine
Tat	HIV-1 Tat basic domain peptide (RKKRRQRRR)
TCSV	Tobacco curly shoot virus
TEM	Transmission electron microscopy
THPMP	(3-trihydroxysilyl)propylmethylphosphonate
TMAPS	N-trimethoxysilylpropyl N,N,N-trimethylammonium chloride
TMV	Tobacco mosaic virus
TPP	Tripolyphosphate
TRITC	Tetramethylrhodamine isothiocyanate
TYLCV	Tomato yellow leaf curl virus
